# Profiling Signal Transduction in Global Marine Biofilms

**DOI:** 10.3389/fmicb.2021.768926

**Published:** 2022-01-07

**Authors:** Ruojun Wang, Weipeng Zhang, Wei Ding, Zhicong Liang, Lexin Long, Wai Chuen Wong, Pei-Yuan Qian

**Affiliations:** ^1^Department of Ocean Science and Hong Kong Branch of Southern Marine Science and Engineering Guangdong Laboratory (Guangzhou), Hong Kong University of Science and Technology, Kowloon, Hong Kong SAR, China; ^2^Southern Marine Science and Engineering Guangdong Laboratory (Guangzhou), Guangzhou, China; ^3^College of Marine Life Sciences, Ocean University of China, Qingdao, China; ^4^Department of Mathematics, Hong Kong University of Science and Technology, Kowloon, Hong Kong SAR, China; ^5^Institute for Advanced Study, Shenzhen University, Shenzhen, China

**Keywords:** signal transduction, marine biofilms, interspecies interactions, microbial community, metagenome

## Abstract

Microbes use signal transduction systems in the processes of swarming motility, antibiotic resistance, virulence, conjugal plasmid transfer, and biofilm formation. However, the signal transduction systems in natural marine biofilms have hardly been profiled. Here we analyzed signal transduction genes in 101 marine biofilm and 91 seawater microbial metagenomes. The abundance of almost all signal transduction-related genes in biofilm microbial communities was significantly higher than that in seawater microbial communities, regardless of substrate types, locations, and durations for biofilm development. In addition, the dominant source microbes of signal transduction genes in marine biofilms were different from those in seawater samples. Co-occurrence network analysis on signal communication between microbes in marine biofilms and seawater microbial communities revealed potential inter-phyla interactions between microorganisms from marine biofilms and seawater. Moreover, phylogenetic tree construction and protein identity comparison displayed that proteins related to signal transductions from Red Sea biofilms were highly similar to those from Red Sea seawater microbial communities, revealing a possible biological basis of interspecies interactions between surface-associated and free-living microbial communities in a local marine environment. Our study revealed the special profile and enrichment of signal transduction systems in marine biofilms and suggested that marine biofilms participate in intercellular interactions of the local ecosystem where they were seeded.

## Introduction

Signal transduction is a stimulus-response process wherein signals are identified and converted into gene activation, which typically results in cellular responses (Bourret and Silversmith, 2010; Gotoh et al., 2010). Microbes use multiple signal transduction systems to respond to environmental changes and mediate diverse physiological processes and intercellular communication, including antibiotic resistance, virulence, symbiotic bacteria-host interactions, and biofilm assemblage (Bassler and Losick, 2006; Boyer and Wisniewski-Dyé, 2009; Gotoh et al., 2010). For example, a two-component system enhances bacitracin resistance in several Gram-positive bacteria, such as *Bacillus subtilis*, *Staphylococcus aureus*, and *Enterococcus faecalis* (Hiron et al., 2011; Dintner et al., 2014; Gebhard et al., 2014), and *N*-acyl homoserine lactone (AHLs) transduction systems are first discovered from marine *Vibrio fischeri* (Milton, 2006) and used as a weapon to compete for survival or to dominate in a natural niche (Defoirdt et al., 2008). Moreover, signal molecules released by microbes trigger population-wide changes in gene expression, promote bacterial colonies to act cooperatively, and facilitate population-dependent adaptive behavior (Atkinson and Williams, 2009; Ng and Bassler, 2009). For example, autoinducer 2 (AI-2) is a non-species-specific signal that regulates intraspecies and interspecies communication among Gram-negative and Gram-positive bacteria (Chen et al., 2002). Cyclic di-GMP (c-di-GMP) decreases flagella-mediated motility, increases bacteria adherence, and enhances biofilm formation (Jenal and Malone, 2006; Wilksch et al., 2011).

Biofilm is a ecological microhabitat where multiple-species microorganisms aggregate, which are often embedded in self-produced extracellular polymeric substances (O’Toole et al., 2000; Flemming and Wingender, 2010; Elias and Banin, 2012). Apart from the planktonic niche, microbes residing in biofilms are highly diverse and in closer proximity to one another, which has inspired the importance of signal transduction (Røder et al., 2016; Zhang et al., 2019). Previous studies have extensively explored the roles of signal transduction systems in the succession of model-species biofilms. For example, nitric-oxide-based transduction induces biofilm formation in various bacteria, such as *V. cholera* and *Legionella pneumophila* (Carlson et al., 2010; Plate and Marletta, 2012). Self-produced exopolysaccharide is recognized as a signal that stimulates biofilm formation in *Pseudomonas aeruginosa* (Irie et al., 2012). *B. subtilis* secretes and involves cyclic di-AMP (c-di-AMP) to facilitate surface-adherence ability (Townsley et al., 2018). In addition, our previous study has expanded the understanding of signal transduction to a natural scenario, in which we treated marine biofilms with different kinds of signal molecules to demonstrate their different effects on microbial structure and function, providing first-hand evidence on the roles of signal transductions in natural biofilm development (Wang et al., 2020). However, when we try to explain the specificity and ecological roles of signal transduction in biofilm microbial communities, poor comprehensive knowledge on the features of signal transduction in natural biofilms holds us back.

In the present study, we profiled the signal transduction systems in 101 global marine biofilm samples developed on different materials immersed in eight locations across the Atlantic, Indian, and Pacific Oceans and then compared the relative abundance with microbial communities in *Tara Oceans* database. Moreover, taxonomic sources of signal transduction genes and interspecies interactions mediated by signal transduction were explored between marine biofilm and neighboring seawater microbial communities.

## Materials and Methods

### Biofilms and Seawater Sampling Across Oceans

During the period of 2013 to 2017, we collected 101 biofilm samples developed on different material surfaces, including Petri dishes, natural rocks, and panels made of zinc, aluminum, titanium, stainless steel, polyether ether ketone, polytetrafluoroethylene, and polyvinyl chloride, and 24 seawater samples from eight locations in the Atlantic Ocean, the Pacific Ocean, the Indian Ocean, and the Red Sea (Zhang et al., 2019). The duration for the development of biofilm samples varied from 3 days to 1 month. Microbes in biofilms were scraped from panels with autoclaved cotton tips. Two liters of seawater was filtered from the adjacent water column where biofilms were developed and filtered through a 0.1 μm membrane filter to collect microbial cells. Microbes from biofilms and seawaters were stored in DNA extraction buffer as described by Zhang et al. (2019). The data sets deposited under Bioproject PRJNA438384 by Zhang et al. (2019) were used in the present study, and the detailed information for biofilm development and adjacent seawater collection is given in Supplementary Figure 1 and Supplementary Table 1.

### DNA Extraction, Sequencing, and Metagenomic Analysis

DNA extraction and sequencing were described in our previous study (Zhang et al., 2019). For DNA sequencing, 350 bp libraries were constructed and sequenced on the Illumina HiSeq X Ten platform in Novogene Bioinformatics Institute (Novogene, Beijing, China) and the HiSeq 2500 System at Beijing Genomics Institute (BGI, Beijing, China). In total, 2.3 terabases of raw reads was generated for all subtidal biofilm metagenomes and adjacent seawater metagenomes. In addition, 67 surface seawater metagenomes were downloaded from *Tara Oceans* (Sunagawa et al., 2015) as a reference for further study.

Quality control of the biofilm and seawater metagenomes was performed using the next-generation sequencing quality control (QC) toolkit (version 2.0; Patel and Jain, 2012). Low-quality reads with a quality score <20 for >30% of the read length were removed. Subsequently, reads from biofilm and seawater samples were assembled into contigs by MEGAHIT (Li et al., 2015) with kmer values of 21–121, which increase in steps of 10.

### Identifying the Abundance of Signal Transduction Genes

To profile the type and abundance of signal transduction genes, metagenomic reads of each sample were mapped to NCBI Nr and Kyoto Encyclopedia of Genes and Genomes (KEGG; Kanehisa et al., 2012) using DIAMOND BLASTx (*e*-value < 1e−7; >60% sequence identity for >60% of the read length) (Buchfink et al., 2015). All the metagenomes were normalized into 10,000,000 reads per sample. The metagenomic reads were then classified on the basis of the SEED category (level 1 SEED functional category classification system was used here) in MEGAN (version 6.9.3) (Huson et al., 2016) and on KEGG PATHWAY Database.^1^ The abundance of a given functional category is indicated by the number of metagenomic reads mapped to genes belonging to its given category. In SEED categories, the mapping results of reads classified into the category “Regulation and Cell Signaling” were selected for subsequent analyses, and the subcategories (i.e., level 2 SEED categories) were visualized. In the KEGG pathway, all the reads mapped to genes belonging to “09132 Signal Transduction” were collected. The significance of dissimilarities related to signal transduction systems between biofilm and seawater microbial communities was tested by two-tailed Student’s *t*-test using the SPSS software package (version 25.0) with relative abundance of the subcategories under “Regulation and Cell Signaling.” Principal coordinates analysis (PCoA) was performed on the basis of the Bray–Curtis distance matrix constructed in PAST (version 2.0) (Hammer et al., 2001).

### Source of Signal Transduction Genes

To identify the source microbes of signal transduction genes, the reads mapped to the “Regulation and Cell Signaling” category (level 2, SEED subcategory) were then mapped to NCBI Nr database by using DIAMOND BLASTx (*e*-value < 1e−7; >60% sequence identity for >60% of the read length) (Buchfink et al., 2015) to identify the taxonomic affiliation. Each resulting read was subsequently classified into relevant phylum level (class level of proteobacteria) in MEGAN (version 6.9.3) (Huson et al., 2016). The contribution of each phylum to the taxonomic difference was assessed using permutational multivariate analysis of variance (PERMANOVA) and SIMPER analysis based on the source phylum composition of all biofilm and seawater microbiota by software PAST (version 2.0) (Hammer et al., 2001).

### Co-occurrence Analysis

Non-random co-occurrence analysis was performed according to the methods described in a previous publication (Freilich et al., 2010). The source phyla of signal transduction genes were used to construct a correlation coefficient matrix in the R studio using the psych (Revelle, 2015) and reshape2 packages (Wickham, 2007). The network of co-occurring phyla was established by setting Spearman’s correlation coefficient at >0.6 and *p*-value at <0.01. Then, the matrix was constructed on the basis of the Benjamini-Hochberg standard false discovery rate correction (FDR-BH) to achieve multiple corrections prior to visualization by the interactive platform Gephi (0.9.2) (Bastian et al., 2009).

### Phylogenetic Analysis

Open reading frames (ORFs) were predicted on the basis of contigs by MetaGeneMark (version 2.8) (Zhu et al., 2010) using the following parameter settings: gene prediction on both strands; gene overlaps were allowed; probability of initiation and termination non-coding state 0.5. Redundant protein sequences (minimum length of 100 bp) derived from the Red Sea biofilm and seawater metagenomes were removed by CD-HIT (>95% sequence identity for >60% of the length of the shorter sequences) (Fu et al., 2012).

Predicted proteins were annotated by COG database (Galperin et al., 2015) based on COG Hidden Markov Models (Dibrova et al., 2017) using HMMER hmmscan (*e*-value < 1e−7) (Finn et al., 2011), and the identified ORFs were assigned to COG categories in “T” function (“T” function in COG: signal transduction). Protein sequences in COG2204 (response regulator containing CheY-like receiver, AAA-type ATPase, and DNA-binding domains) were selected as the reference, and the genes mapped to this category were used to construct the phylogenetic tree with ClustW method and 1,000 bootstraps by MEGA 6 (Tamura et al., 2013).

### Percentage Identity of Signal Transduction Genes in Biofilms Compared With Those in Seawater

Open reading frames from the Red Sea biofilm and seawater metagenomes were identified as the signal transduction genes in the COG database. The biofilm-derived proteins were then compared with seawater-derived ones by Blastp (*e*-value < 1e−7; >60% sequence identity for >60% of the read length) to identify top alignments (Johnson et al., 2008). The generated pairs over 70% identity and 100 bp alignment were selected as signal transduction genes of high similarity.

## Results

### Metagenomic Profile of Signal Transduction Genes in Marine Biofilm and Seawater Microbial Communities

**FIGURE 1:**
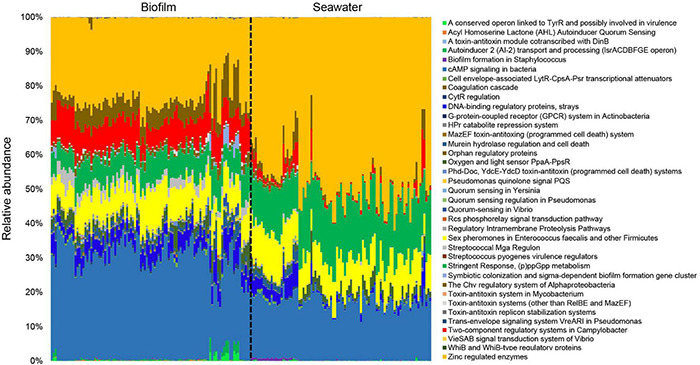
Overall profiles of signal transduction genes in 101 marine biofilm and 91 seawater samples. The biofilms were collected in eight different locations around the world oceans. The relative abundance of different SEED functional categories (SEED2 level) that are related to microbial signal transduction is given. All the metagenomes were normalized to equal data size (i.e., 10,000,000 reads per metagenome) before performing the analysis.

The profile of signal transduction genes in the marine environment was presented based on metagenomic tools (Figure 1). In the metagenomes of 101 biofilm samples and 91 seawater samples, the visualized signal transduction and regulation profiles at level 2 SEED functional classification were divided into 42 categories under “Regulation and Cell Signaling” (Figure 1). These categories included several quorum sensing (QS) gene families (e.g., QS in *Vibrio*, QS regulation in *Pseudomonas*, QS in *Yersinia*, AI-2 transporters, and AHL inducers), two-component regulator families (e.g., two-component regulatory systems in *Campylobacter*, oxygen and light sensor PpaA-PpsR, and mazE-mazF system), and unknown regulatory genes related to biofilm formation (e.g., biofilm formation in *Staphylococcus*) and virulence (e.g., *Streptococcus* pyogenes virulence regulators). The composition and relative abundance of these signal transduction categories displayed distinct patterns between the biofilm and seawater microbial communities (Figure 1), indicating the specific profile of signal transduction systems in marine biofilms.

**FIGURE 2:**
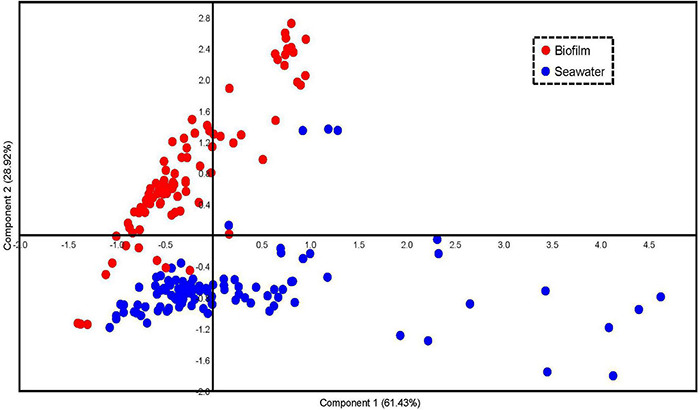
Principal coordinates analysis (PCoA) of regulation and signal transduction subsystems in biofilm and seawater microbial communities. In total, 101 normalized biofilm metagenomes and 91 normalized seawater metagenomes were included in this analysis. Bray–Curtis distances were calculated based on the type and relative abundance of regulation and signal transduction subsystems and visualized in the PCoA plot.

Principal coordinates analysis based on the “Regulation and Cell Signaling” (subsystems and abundances) was conducted to confirm the differentiation of the signal transduction genes in the marine environment, which clearly separated the biofilms and seawater samples in the plot (Figure 2). Moreover, all the biofilm samples were clustered in a group, whereas the seawater samples were scattered (Figure 2), indicating smaller variations of signal transduction genes in biofilm microbial communities.

### Enriched Signal Transduction Genes in Marine Biofilms

**FIGURE 3:**
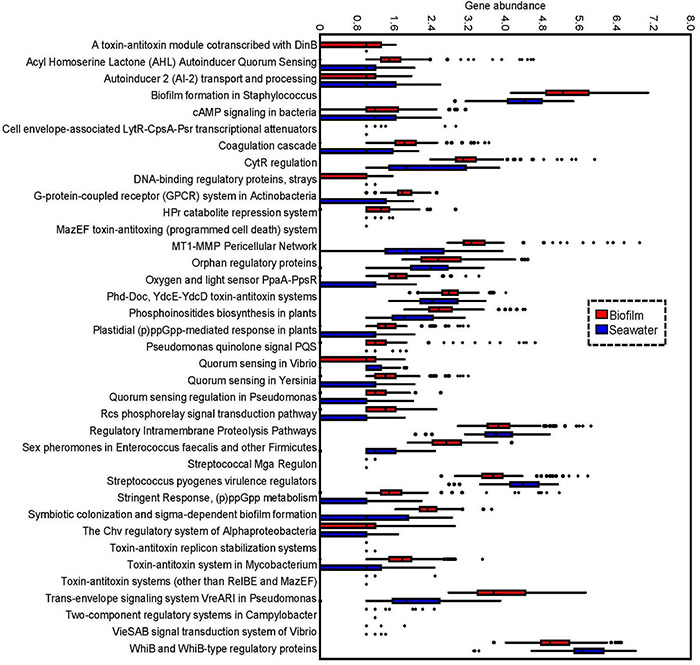
Difference of regulation and signal transduction subsystems between the biofilm and seawater microbial communities. The abundance (based on fourth root) of different subsystems under the category. Regulation and signal transduction in 101 biofilms and 91 seawater metagenomes were compared, and the 37 significantly different (two-tailed Student’s *t*-test, *p* < 0.01) subsystems are shown. All the metagenomes were normalized to equal data size (i.e., 10,000,000 reads per metagenome), and the abundance of a subsystem was indicated by the number of metagenomic reads mapped to the gene belonging to this subsystem.

To further explore features of the signal transduction in marine biofilms, statistical analyses were performed to identify the abundances of signal transduction gene categories between the global biofilm and seawater samples. Most genes in the “Regulation and Cell Signaling” categories of SEED were more abundant in biofilms than those in seawater (two-tailed Student’s *t*-test, *p* < 0.01). A total of 35 subsystems were significantly (*p* < 0.01) enriched in the biofilm samples, whereas only two were significantly enriched in the seawater samples (Figure 3). Notably, almost all the genes related to QS were concentrated in biofilms. For example, the genes that belonged to the AHL autoinducer category were abundant (with up to 4,000 reads per biofilm metagenome), but almost no reads could be mapped by the seawater metagenomes (Figure 3). By contrast, the gene categories of G-protein-coupled receptor systems in Actinobacteria and of WhiB and WhiB-type regulatory proteins were enriched in the seawater samples (Figure 3). To confirm these results, we counted the number of reads that were mapped to “09132 Signal transduction” categories according to KEGG pathway classification. We found that only the HIF-1 signaling pathway was enriched in the seawater samples, whereas almost all signaling pathways were enriched in the biofilm samples (Supplementary Figure 2). Furthermore, the abundance of these genes varied slightly in biofilms but dramatically in seawater microbial communities (Figure 3), which was consistent with the results of PCoA (Figure 2).

### Source of Signal Transduction Genes

**FIGURE 4:**
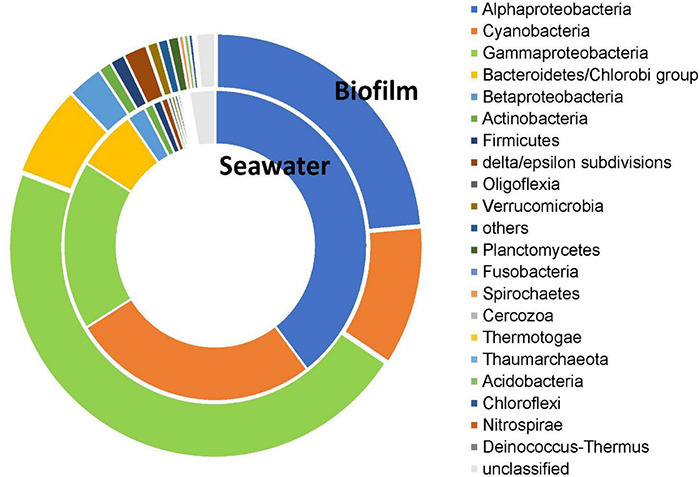
Distribution of source microbes of signal transduction genes in 101 biofilms and 91 seawater samples. The taxonomic affiliation of signal transduction genes was determined in the phylum level (class of proteobacteria) of each sample, and the average of source phyla in the biofilms and seawater microbial communities was calculated, respectively to generate the taxonomic affiliation pattern. In the biofilm microbial communities, Gammaproteobacteria contributed most signal transduction genes, whereas the most contributed bacteria were Alphaproteobacteria.

To understand the source of signal transduction genes, we determined the taxonomic affiliation of these genes in biofilm and seawater. The results demonstrated that dominant source phyla (here and after, Proteobacteria fallen into the class level) in biofilm and seawater were different, which could be confirmed by PERMANOVA test (*p* < 0.001). In the biofilm microbial communities, Gammaproteobacteria contributed 46.38% signal transduction genes, followed by Alphaproteobacteria (23.58%) and Cyanobacteria (10.71%), whereas among microbes in seawater, Alphaproteobacteria had the most contribution (39.66%), followed by Cyanobacteria (26.49%) and Gammaproteobacteria (17.71%; Figure 4). SIMPER analysis unveiled the top five dominant source phyla that contributed to the taxonomic differentiation of signal transduction genes, accounting for 85.73% taxonomic difference between biofilm and seawater samples (Supplementary Figure 3).

### Potential Signal Transduction Between the Red Sea Biofilm and Adjacent Seawater Microbial Communities

**FIGURE 5:**
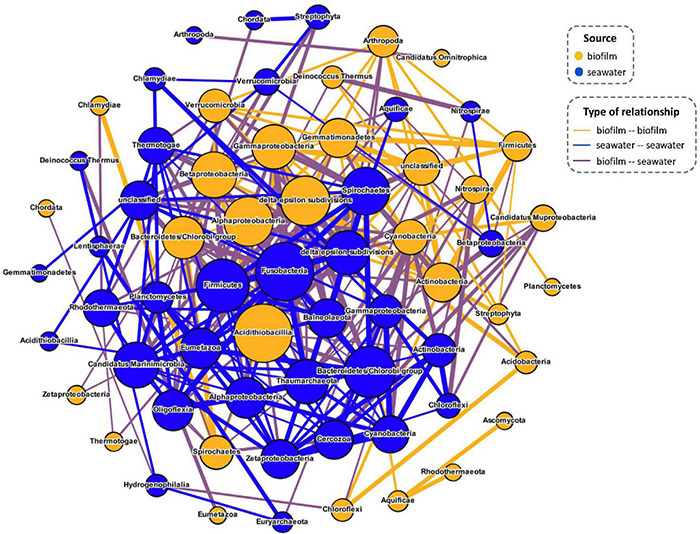
Co-occurrence network of signal transduction gene source phyla (classes of proteobacteria) in the Red Sea biofilms and seawater microbial communities. The size of each node is proportional to its number of connections, and the thickness of lines represents the absolute value of correlation coefficient.

Considering that the microbial community of biofilm seeded by surrounding seawater is not an insular and enclosed ecosystem in the marine environment, the signal transduction to connect the interspecies interactions of microbes from biofilm and neighboring seawater is important. Therefore, 12 biofilm samples and 12 seawater samples collected in pairs from the Red Sea were selected for the following analyses. The co-occurrence network was constructed based on all source phyla of signal transduction genes to explore the interspecies interactions between biofilms and adjacent seawater. Among the 331 potential correlations identified, 137 inter-phyla interactions between the Red Sea biofilm communities and their adjacent seawater communities were identified (Figure 5). In Red Sea biofilms, 30 phyla were involved in the inter-phyla relationships, of which 25 phyla could achieve inter-phyla correlations with the 31 phyla derived from adjacent seawater samples (Supplementary Figure 4). Acidithiobacillia contributed the most connections in biofilms, followed by Alphaproteobacteria and Deltaproteobacteria/Epsilon subdivisions, whereas Fusobacteria, Firmicutes, and Bacteroidetes/Chlorobi were found in seawater (Figure 5).

**FIGURE 6:**
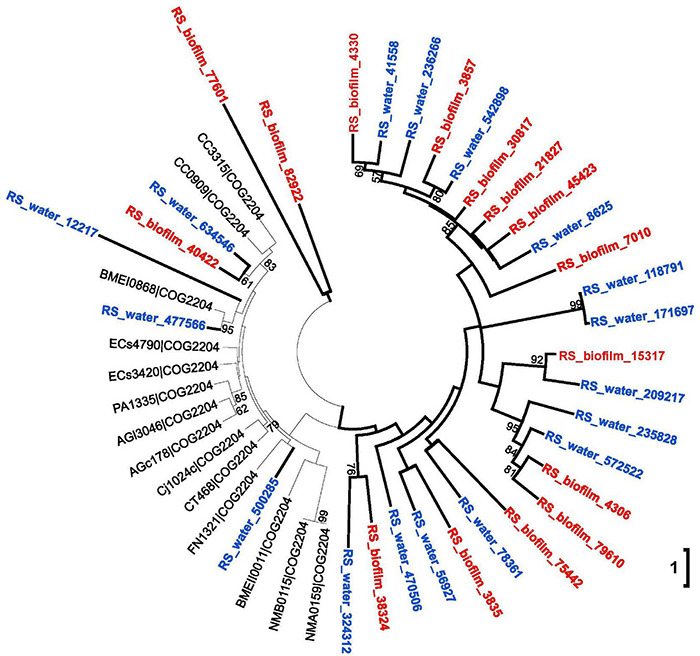
Phylogenetic tree of the response regulator subunit COG2204 identified from the Red Sea biofilm and seawater microbial communities. The protein sequences that could be aligned by ClustalW were used to construct a maximum likelihood tree with 1,000 replicates. Bootstrap values (>50) are shown on the branches. The independent branch indicated the high sequence similarity between proteins from biofilm and seawater samples.

We hypothesized that if biofilm and adjacent seawater communities shared high sequence similarity of substantial signal transduction genes, the related signal transduction systems could be employed and responded by microbes both from marine biofilms and neighboring planktonic counterparts. To test the hypothesis, the sequence similarity of signal transduction genes between Red Sea biofilm and the nearby seawater samples were identified. There were 1,74,273 protein sequences in Red Sea biofilm samples (*n* = 12) annotated as signal transduction genes by COG database, and a total of 15,780 (9.05%) protein sequences shared over 70% identity and 100 bp alignment with those from the seawater samples (*n* = 12), which could be considered as highly similar signal transduction genes (Supplementary Figure 5). To confirm the biological basis of interspecies signaling interactions between surface-associated and free-living microbial communities, a phylogenetic tree of a protein ortholog was established. The proteins assigned to a subunit COG2204 (response regulator containing CheY-like receiver, AAA-type ATPase, and DNA-binding domains) of a two-component transduction system were selected and the phylogenetic tree showed a relatively independent branch for the response regulator proteins from the Red Sea biofilm and seawater (Figure 6), indicating the high protein similarity of signal transduction genes between marine biofilms and adjacent seawater.

## Discussion

Signal transduction is an important regulatory system and plays a key role in biofilm formation and development (Guttenplan et al., 2010; Beauregard et al., 2013; Mielich-Süss and Lopez, 2015; Armbruster and Parsek, 2018). Signal transduction genes, particularly the QS genes, are important for various microbial activities, such as symbiosis, virulence, competence, conjugation, antibiotic production, motility, sporulation, and biofilm formation (Miller and Bassler, 2001). In the present study, we investigated the features of signal transduction in marine environment and found substantial differences in both the composition and abundance of signal transduction systems between the marine biofilm and seawater microbial communities.

Marine environment is an ideal natural ecosystem to explore the collective features of signal transduction systems in biofilm-associated microbial communities compared with free-living microbes. Here, we determined the differences in the patterns of signal transduction systems in biofilm and planktonic bacteria. Since the microbial community of marine biofilms varies with the type of substrate, location, temporal changes and other environmental factors (Oberbeckmann et al., 2016; Zhang et al., 2016; Antunes et al., 2020), the fluctuations among global biofilms are expected. However, our results manifested the smaller variation of the signal transduction profile among global marine biofilm in comparison with that in seawater, indicating surface-associated microorganisms in more stable microenvironments can escape from the worldwide displacement with seawater flow (Lewis, 2001). In previous studies, it has been revealed that biofilms were closely interconnected with signal transduction especially QS system in model species (Ng and Bassler, 2009; Solano et al., 2014). For example, the relationship between the biofilm differentiation process of *P. aeruginosa* and cell-cell signaling system LasI/LasR was firstly evidenced in the 1990s (Davies et al., 1998); autoinducer-receptor pairs could control the biofilm development of *V. cholerae* (Papenfort et al., 2017) and increased c-di-GMP could facilitate the exopolysaccharide production, cell-to-cell aggregation, and biofilm formation in *Burkholderia pseudomallei* (Lee et al., 2010). In the present study, the importance of signal transduction in biofilms was emphasized from a natural perspective. Our findings indicated that several categories of signal transduction genes were enriched in marine biofilms, providing direct evidence for possible correlation between signal transduction and microbial life in multi-species community and suggesting the important roles of signal transduction in the niche differentiation processes between the free-living and surface-associated microbes (Hmelo, 2017). Furthermore, almost all the well-studied genes of the AHL-dependent QS system, *Pseudomonas quinolone* signal transduction system, and AI-2 signal transduction system were found to be enriched in marine biofilms as discovered in the present study, by metagenomic analysis, highlighting the significance of QS genes, as a special type of signal transduction genes, to the marine biofilms.

The source microbes of signal transduction systems have been investigated by several studies. For example, Fernández-Gomez et al. (2013) have raised that two-component systems were the largest functional families among four marine Bacteroidetes; proteobacteria use AHLs to regulate multiple physiological processes and group activities (Nakashima et al., 2006). Our present study profiled the source microbes of signal transduction genes in global marine environment and indicated the dominance of proteobacteria among the source phyla of signal transduction systems. Here we found that Gammaproteobacteria was the most important source of signal transduction genes in our global marine biofilm samples, whereas Alphaproteobacteria contributed most signal transduction genes in the seawater samples. Notably, these two bacterial taxa were the most predominant microbial composition in marine biofilms and seawater, respectively (Zhang et al., 2019). In our early laboratory investigation, correlatively, we found that bacterial genera with signal transduction genes in biofilms could obtain high abundance when the microbial community was treated by signal molecules (Wang et al., 2020). Collectively, these results suggested that the abundant species occupied main positions in signal communication of bacterial groups, indicating that enhanced signal transduction systems may help microbes take advantage in a competitive multi-microorganism environment.

Although signal transduction is associated with microbial assemblage, however, natural marine biofilm is not an independent microecosystem, in which microorganisms are seeded by and dispersed into the adjacent free-living counterpart. Therefore, signal transduction could act as a bridge linking the communication between the microbes from marine biofilms with those in adjacent seawater. In the present study, our results bridged the understanding gap of inter-phyla signal communications between surface-associated and free-living microbial communities and revealed some biological bases of signal communications in the marine environment. On one hand, multiple correlations on source phyla of signal transduction genes were examined, extending previous findings in model species to natural microbial communities. For example, *luxS* orthologs encoding a putative interspecies signal molecule AI-2 were identified in many microbes, including Firmicutes, Epsilonproteobacteria, Gammaproteobacteria, and Betaproteobacteria (Bodor et al., 2008); in the current study, signal transduction connections linking biofilm-derived Betaproteobacteria with Firmicutes, Epsilonproteobacteria, and Gammaproteobacteria from seawater could be identified in current co-occurrence analysis. Moreover, Stoodley et al. (2002) raised the possibility of interspecies interactions in natural biofilms; current co-occurrence analysis also demonstrated the inter-phyla signal transduction in natural biofilms, providing a possible bioinformatic solution to uncover the interactions of microorganisms residing closely together in biofilms as reported by Røder et al. (2016). On the other hand, the similarity identification of protein sequence and phylogenetic analysis showed a high sequence similarity of substantial signal transduction genes between Red Sea biofilm and seawater samples, implying some signaling regulatory systems could be employed by both the natural biofilm and neighboring microbial communities on the basis of which possible interactions could occur. However, given the difficulty in identifying the actual interspecies communication in the biofilm and adjacent seawater, exact inter-phyla interactions *via* signal transduction in natural environments remain to be explored.

Our present study has drawn an overall picture of signal transduction in the marine environment, demonstrated enriched signal transduction genes, and determined distinct taxonomic sources in marine biofilms, providing first-hand evidence of the special profile of signal transduction systems in natural biofilms. Meanwhile, the communication platform constructed by signal transduction in microbes of biofilms and seawater has been clarified, emphasizing the interspecies interactions between surface-associated communities and free-living counterpart in the marine environment. Collectively, our findings infer that signal transduction not only shows the special profile and enrichment in marine biofilms but serves as an important link of marine biofilms to the surrounding environment, both of which help achieve their roles in the biofilm niche differentiation from seawater.

## Data Availability Statement

The metagenomic data for biofilm and seawater samples are deposited as Bioproject PRJNA438384 in NCBI, and detailed sampling information was summarized in Supplementary Table 1.

## Author Contributions

P-YQ and WZ conceptualized this study. RW, WZ, WD, and WW collected the samples. RW, WD, and ZL performed the analysis. LL and ZL provided the biochemical and bioinformatic technical support. RW and P-YQ wrote the manuscript. All authors contributed to the article and approved the submitted version.

## Conflict of Interest

The authors declare that the research was conducted in the absence of any commercial or financial relationships that could be construed as a potential conflict of interest.

## Publisher’s Note

All claims expressed in this article are solely those of the authors and do not necessarily represent those of their affiliated organizations, or those of the publisher, the editors and the reviewers. Any product that may be evaluated in this article, or claim that may be made by its manufacturer, is not guaranteed or endorsed by the publisher.

## Footnotes

1
https://www.genome.jp/kegg/pathway.html
