# Supplementary material for: Profiling Signal Transduction in Global Marine Biofilms
Source: Front Microbiol. 2022 Jan 7;12:768926. doi: 10.3389/fmicb.2021.768926 (PMC8776716; doi:10.3389/fmicb.2021.768926)
Supplement: Supplementary file 1 [file Data_Sheet_1.PDF]

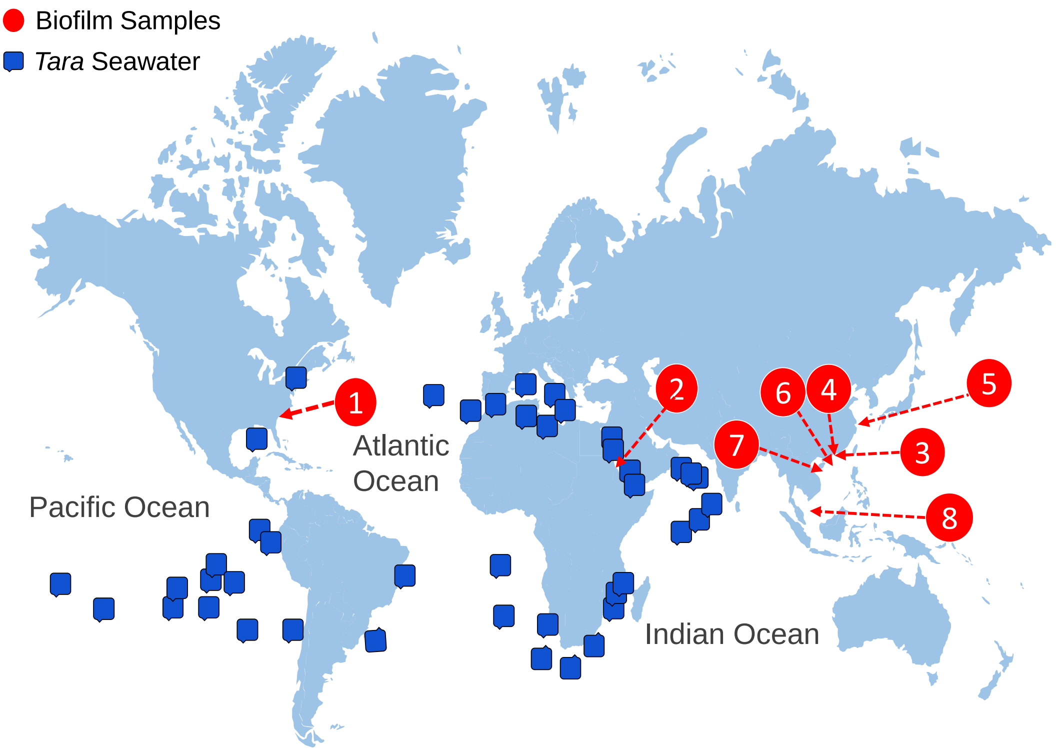

Supplementary Figure S1 Sampling locations in the present study. The eight locations of samples collection as follow: (1) South Atlantic, (2) Red Sea, (3) Hong Kong Water, (4) Yung Shu O Bay, (5) East China Sea, (6) South China Sea 1, (7) South China Sea 2, and (8) South China Sea 3. Tara surface seawater samples used as reference are indicated. This figure was modified from Fig. S1 in Zhang et al. 2019 to indicate the sample locations in the present study.

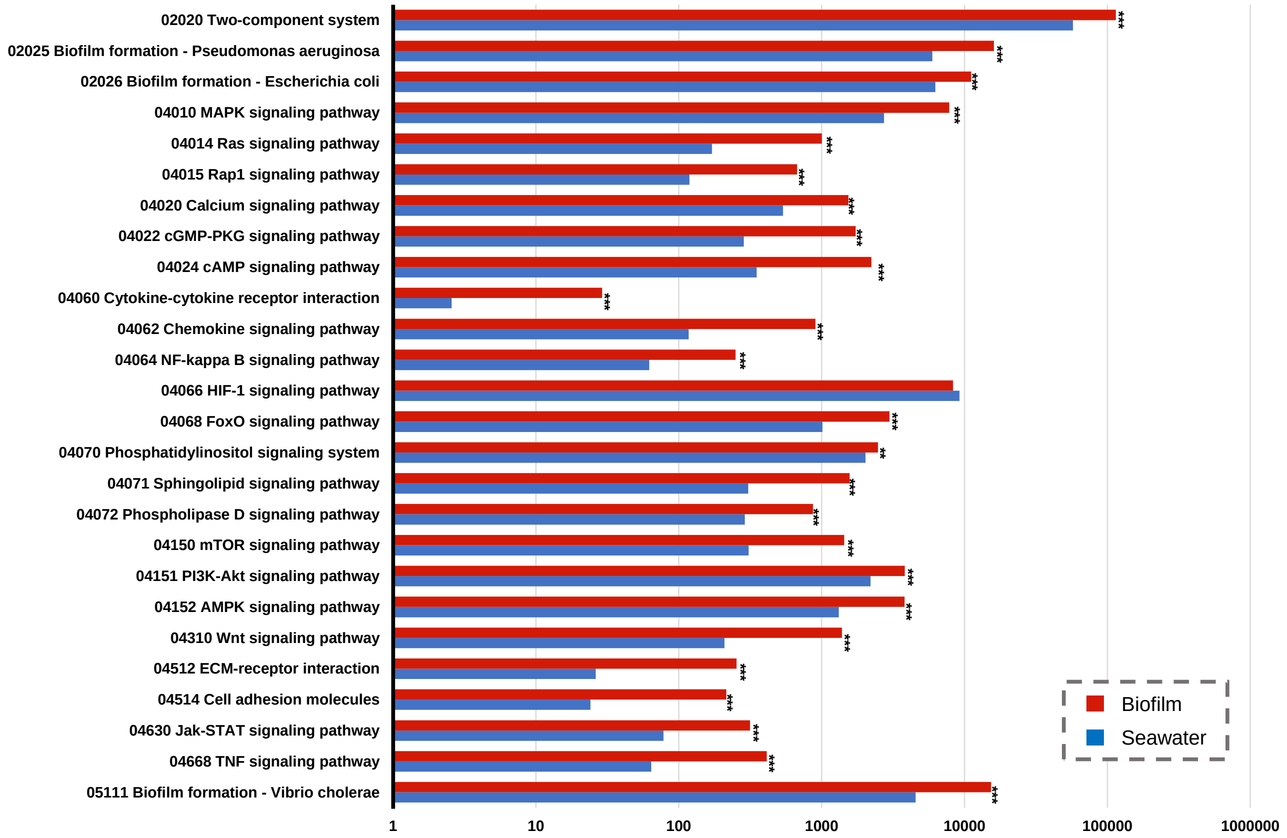

Supplementary Figure S2 Difference of signal transduction pathways between the biofilm and seawater microbial communities. The abundance of all subsystems under the category '09132 Signal transduction' of KEGG pathway in 101 biofilms and 91 seawater metagenomes is shown. All metagenomes were normalized to equal data size (i.e., 10,000,000 reads per metagenome), and the abundance of a subsystem was indicated by the number of metagenomic reads mapped to the gene belonging to this subsystem (two-tailed Student's t-test; **, p-value <0.01; ***, p-value <0.001).

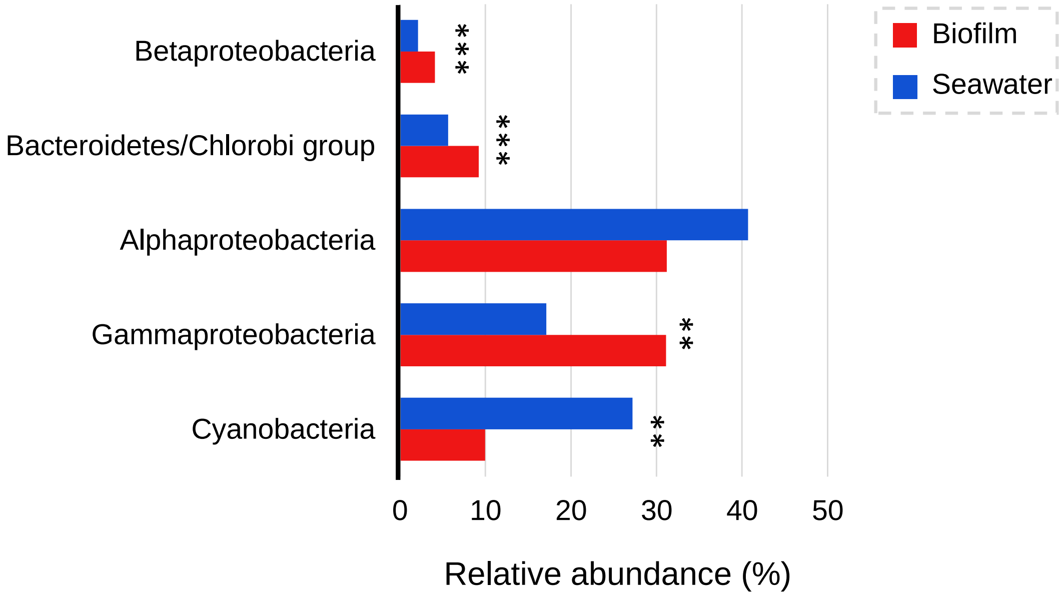

Supplementary Figure S3 Top 5 contributed phyla (class of proteobacteria) to the difference of taxonomic affiliation between biofilms and seawater. Statistical analysis was performed based on their relative abundance (two-tailed Student's t-test; **, p-value <0.01; ***, p-value <0.001).

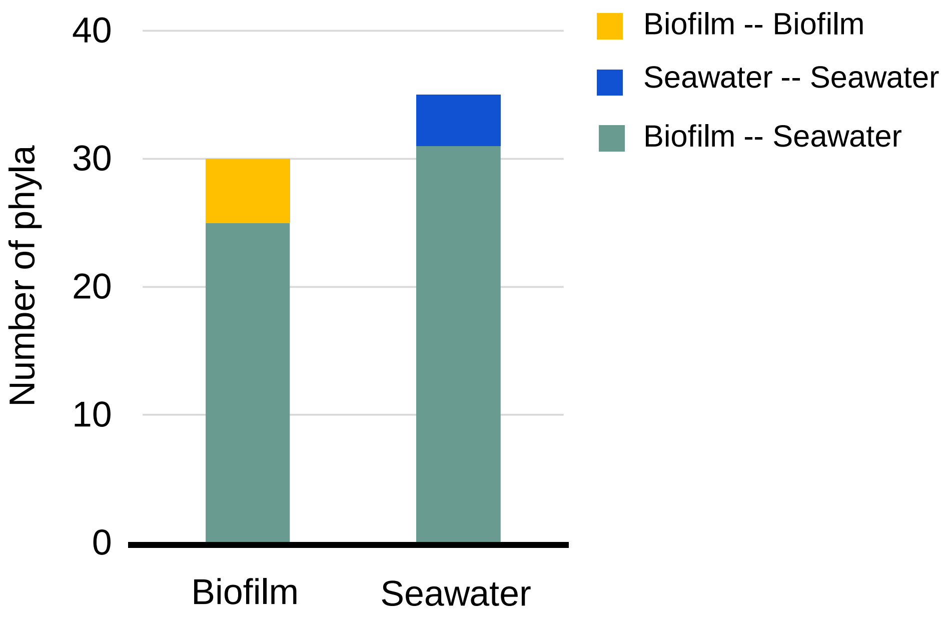

Supplementary Figure S4 Type distribution of interphylum interactions in Red Sea biofilm and seawater samples. The interspecies interactions were estimated by co-occurrence analysis based on the source phyla of signal transduction genes.

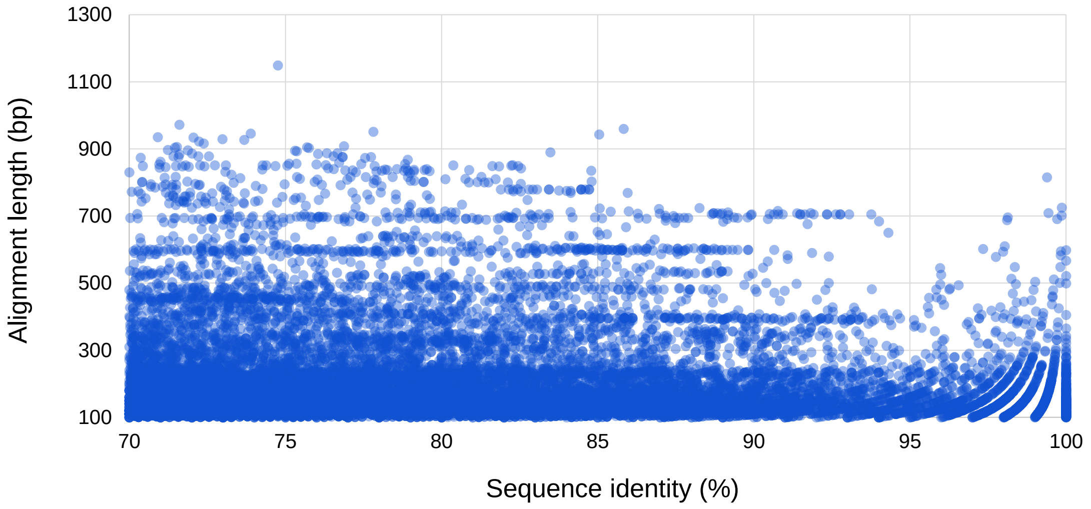

Supplementary Figure S5 Similarity of signal transduction genes identified from Red Sea biofilms and seawater. The biofilm-derived signal transduction genes were BLASTp searched against signal transduction genes from seawater. The BLASTp hits with over 70% identity and more than 100 bp alignments were shown.

Supplementary Table S1 Information of the metagenomes of the 101 biofilm and 91 seawater samples collected in marine environments. The biofilms were developed in subtidal zones of eight different locations on a variety of materials.

**Biofilm samples (Current study).**								
**Sample No.**	**Biofilm metagenomes**	**Total sequences (×2)**	**Sampling locations**	**Sampling time**	**Biofilm substrata**	**Durations for biofilm development**	**Sequencing center**	**Accession (in NCBI)**
1	B-USA1	79551308	Coastal water of the Sapelo Island (31.42, -81.30)	NOV, 2017	Petri dish	28 days	Novogene	Bioproject PRJNA438384
2	B-USA2	84089314	Coastal water of the Sapelo Island (31.42, -81.30)	NOV, 2017	Petri dish	28 days	Novogene	Bioproject PRJNA438384
3	B-USA3	56689852	Coastal water of the Sapelo Island (31.42, -81.30)	NOV, 2017	Petri dish	28 days	Novogene	Bioproject PRJNA438384
4	B-RSA1	62353198	Red Sea (22.20, 39.03)	APR, 2016	Zinc panel	30 days	BGI	Bioproject PRJNA438384
5	B-RSA2	55621490	Red Sea (22.20, 39.03)	APR, 2016	Zinc panel	30 days	BGI	Bioproject PRJNA438384
6	B-RSA3	51602709	Red Sea (22.20, 39.03)	APR, 2016	Zinc panel	30 days	BGI	Bioproject PRJNA438384
7	B-RSB1	58257555	Red Sea (22.20, 39.04)	APR, 2016	Zinc panel	30 days	BGI	Bioproject PRJNA438384
8	B-RSB2	57796955	Red Sea (22.20, 39.04)	APR, 2016	Zinc panel	30 days	BGI	Bioproject PRJNA438384

9	B-RSB3	63412812	Red Sea (22.20, 39.04)	APR, 2016	Zinc panel	30 days	BGI	Bioproject PRJNA438384
10	B-RSC1	56077440	Red Sea (22.20, 39.05)	APR, 2016	Zinc panel	30 days	BGI	Bioproject PRJNA438384
11	B-RSC2	54513891	Red Sea (22.20, 39.05)	APR, 2016	Zinc panel	30 days	BGI	Bioproject PRJNA438384
12	B-RSC3	63510497	Red Sea (22.20, 39.05)	APR, 2016	Zinc panel	30 days	BGI	Bioproject PRJNA438384
13	B-RSiii1	54288177	Red Sea (22.20, 39.04)	JUN, 2016	Zinc panel	30 days	BGI	Bioproject PRJNA438384
14	B-RSiii2	56176432	Red Sea (22.20, 39.04)	JUN, 2016	Zinc panel	30 days	BGI	Bioproject PRJNA438384
15	B-RSiii3	43683295	Red Sea (22.20, 39.04)	JUN, 2016	Zinc panel	30 days	BGI	Bioproject PRJNA438384
16	B-HK13071	48941596	Hong Kong Water (22.34, 114.27)	JUL, 2013	Petri dish	12 days	Novogene	Bioproject PRJNA438384
17	B-HK13072	40492706	Hong Kong Water (22.34, 114.27)	JUL, 2013	Petri dish	12 days	Novogene	Bioproject PRJNA438384
18	B-HK13073	41043436	Hong Kong Water (22.34, 114.27)	JUL, 2013	Petri dish	12 days	Novogene	Bioproject PRJNA438384
19	B-HK14071	60032585	Hong Kong Water (22.34, 114.27)	JUL, 2014	Petri dish	12 days	Novogene	Bioproject PRJNA438384
20	B-HK14072	67332696	Hong Kong Water (22.34, 114.27)	JUL, 2014	Petri dish	12 days	Novogene	Bioproject PRJNA438384

21	B-HK14073	59375875	Hong Kong Water (22.34, 114.27)	JUL, 2014	Petri dish	12 days	Novogene	Bioproject PRJNA438384
22	B-HK15041	31863978	Hong Kong Water (22.34, 114.27)	APR, 2015	Petri dish	12 days	Novogene	Bioproject PRJNA438384
23	B-HK15042	41513751	Hong Kong Water (22.34, 114.27)	APR, 2015	Petri dish	12 days	Novogene	Bioproject PRJNA438384
24	B-HK15043	30850236	Hong Kong Water (22.34, 114.27)	APR, 2015	Petri dish	12 days	Novogene	Bioproject PRJNA438384
25	B-HK15051	34908917	Hong Kong Water (22.34, 114.27)	MAY, 2015	Petri dish	12 days	Novogene	Bioproject PRJNA438384
26	B-HK15052	37980801	Hong Kong Water (22.34, 114.27)	MAY, 2015	Petri dish	12 days	Novogene	Bioproject PRJNA438384
27	B-HK15053	43420017	Hong Kong Water (22.34, 114.27)	MAY, 2015	Petri dish	12 days	Novogene	Bioproject PRJNA438384
28	B-HK15071	29289682	Hong Kong Water (22.34, 114.27)	JUL, 2015	Petri dish	12 days	Novogene	Bioproject PRJNA438384
29	B-HK15072	23326259	Hong Kong Water (22.34, 114.27)	JUL, 2015	Petri dish	12 days	Novogene	Bioproject PRJNA438384
30	B-HK15073	37218325	Hong Kong Water (22.34, 114.27)	JUL, 2015	Petri dish	12 days	Novogene	Bioproject PRJNA438384
31	B-HK15091	34434692	Hong Kong Water (22.34, 114.27)	SEP, 2015	Petri dish	12 days	Novogene	Bioproject PRJNA438384
32	B-HK15092	48091179	Hong Kong Water (22.34, 114.27)	SEP, 2015	Petri dish	12 days	Novogene	Bioproject PRJNA438384

33	B-HK16041	47619925	Hong Kong Water (22.34, 114.27)	APR, 2016	Petri dish	12 days	Novogene	Bioproject PRJNA438384
34	B-HK16042	50145495	Hong Kong Water (22.34, 114.27)	APR, 2016	Petri dish	12 days	Novogene	Bioproject PRJNA438384
35	B-HK16043	47252685	Hong Kong Water (22.34, 114.27)	APR, 2016	Petri dish	12 days	Novogene	Bioproject PRJNA438384
36	B-HK16051	56226646	Hong Kong Water (22.34, 114.27)	MAY, 2016	Petri dish	12 days	Novogene	Bioproject PRJNA438384
37	B-HK16052	47120035	Hong Kong Water (22.34, 114.27)	MAY, 2016	Petri dish	12 days	Novogene	Bioproject PRJNA438384
38	B-HK16053	46636183	Hong Kong Water (22.34, 114.27)	MAY, 2016	Petri dish	12 days	Novogene	Bioproject PRJNA438384
39	B-HK16061	48344213	Hong Kong Water (22.34, 114.27)	JUN, 2016	Petri dish	12 days	Novogene	Bioproject PRJNA438384
40	B-HK16062	50189837	Hong Kong Water (22.34, 114.27)	JUN, 2016	Petri dish	12 days	Novogene	Bioproject PRJNA438384
41	B-HK16063	52421535	Hong Kong Water (22.34, 114.27)	JUN, 2016	Petri dish	12 days	Novogene	Bioproject PRJNA438384
42	B-HK16071	47707626	Hong Kong Water (22.34, 114.27)	JUL, 2016	Petri dish	12 days	Novogene	Bioproject PRJNA438384
43	B-HK16072	54090458	Hong Kong Water (22.34, 114.27)	JUL, 2016	Petri dish	12 days	Novogene	Bioproject PRJNA438384
44	B-HK16073	50668275	Hong Kong Water (22.34, 114.27)	JUL, 2016	Petri dish	12 days	Novogene	Bioproject PRJNA438384

45	B-HK16081	46399209	Hong Kong Water (22.34, 114.27)	AUG, 2016	Petri dish	12 days	Novogene	Bioproject PRJNA438384
46	B-HK16082	56530392	Hong Kong Water (22.34, 114.27)	AUG, 2016	Petri dish	12 days	Novogene	Bioproject PRJNA438384
47	B-HK16083	42721652	Hong Kong Water (22.34, 114.27)	AUG, 2016	Petri dish	12 days	Novogene	Bioproject PRJNA438384
48	B-HK16091	52696257	Hong Kong Water (22.34, 114.27)	SEP, 2016	Petri dish	12 days	Novogene	Bioproject PRJNA438384
49	B-HK16092	62703270	Hong Kong Water (22.34, 114.27)	SEP, 2016	Petri dish	12 days	Novogene	Bioproject PRJNA438384
50	B-HK16093	51060810	Hong Kong Water (22.34, 114.27)	SEP, 2016	Petri dish	12 days	Novogene	Bioproject PRJNA438384
51	B-HK17061	97833514	Hong Kong Water (22.34, 114.27)	JUN, 2017	Petri dish	12 days	Novogene	Bioproject PRJNA438384
52	B-HK17062	77261144	Hong Kong Water (22.34, 114.27)	JUN, 2017	Petri dish	12 days	Novogene	Bioproject PRJNA438384
53	B-HK17063	69787326	Hong Kong Water (22.34, 114.27)	JUN, 2017	Petri dish	12 days	Novogene	Bioproject PRJNA438384
54	B-HK17071	39326525	Hong Kong Water (22.34, 114.27)	JUL, 2017	Petri dish	12 days	Novogene	Bioproject PRJNA438384
55	B-HK17072	52976823	Hong Kong Water (22.34, 114.27)	JUL, 2017	Petri dish	12 days	Novogene	Bioproject PRJNA438384
56	B-HK17073	53046204	Hong Kong Water (22.34, 114.27)	JUL, 2017	Petri dish	12 days	Novogene	Bioproject PRJNA438384

57	B-HK17091	42602261	Hong Kong Water (22.34, 114.27)	SEP, 2017	Petri dish	12 days	Novogene	Bioproject PRJNA438384
58	B-HK17092	39917307	Hong Kong Water (22.34, 114.27)	SEP, 2017	Petri dish	12 days	Novogene	Bioproject PRJNA438384
59	B-HK17093	40607317	Hong Kong Water (22.34, 114.27)	SEP, 2017	Petri dish	12 days	Novogene	Bioproject PRJNA438384
60	B-HK17111	30735939	Hong Kong Water (22.34, 114.27)	NOV, 2017	Petri dish	12 days	Novogene	Bioproject PRJNA438384
61	B-HK17112	28899285	Hong Kong Water (22.34, 114.27)	NOV, 2017	Petri dish	12 days	Novogene	Bioproject PRJNA438384
62	TIANamp1	68171173	Hong Kong Water (22.34, 114.27)	DEC, 2017	Petri dish	12 days	Novogene	Bioproject PRJNA438384
63	TIANamp2	71369150	Hong Kong Water (22.34, 114.27)	DEC, 2017	Petri dish	12 days	Novogene	Bioproject PRJNA438384
64	AllPrep1	100877758	Hong Kong Water (22.34, 114.27)	DEC, 2017	Petri dish	12 days	Novogene	Bioproject PRJNA438384
65	AllPrep2	85715477	Hong Kong Water (22.34, 114.27)	DEC, 2017	Petri dish	12 days	Novogene	Bioproject PRJNA438384
66	DNeasy1	98183244	Hong Kong Water (22.34, 114.27)	DEC, 2017	Petri dish	12 days	Novogene	Bioproject PRJNA438384
67	DNeasy2	76843782	Hong Kong Water (22.34, 114.27)	DEC, 2017	Petri dish	12 days	Novogene	Bioproject PRJNA438384
68	C1	53580672	Hong Kong Water (22.34, 114.27)	DEC, 2017	Petri dish	12 days	Novogene	Bioproject PRJNA438384

69	C2	25643083	Hong Kong Water (22.34, 114.27)	DEC, 2017	Petri dish	12 days	Novogene	Bioproject PRJNA438384
70	AL1	48555539	Hong Kong Water (22.34, 114.27)	MAR, 2017	Aluminium panel	30 days	Novogene	Bioproject PRJNA438384
71	AL2-P2	47404368	Hong Kong Water (22.34, 114.27)	MAR, 2017	Aluminium panel	30 days	Novogene	Bioproject PRJNA438384
72	PEEK1	47718960	Hong Kong Water (22.34, 114.27)	MAR, 2017	Poly(ether-ether-ketone) panel	30 days	Novogene	Bioproject PRJNA438384
73	PEEK2	49791642	Hong Kong Water (22.34, 114.27)	MAR, 2017	Poly(ether-ether-ketone) panel	30 days	Novogene	Bioproject PRJNA438384
74	PTFE1	42750301	Hong Kong Water (22.34, 114.27)	MAR, 2017	Polytetrafluroethylene panel	30 days	Novogene	Bioproject PRJNA438384
75	PTFE2	44444811	Hong Kong Water (22.34, 114.27)	MAR, 2017	Polytetrafluroethylene panel	30 days	Novogene	Bioproject PRJNA438384
76	PVC1	52468172	Hong Kong Water (22.34, 114.27)	MAR, 2017	Poly (vinyl chloride) panel	30 days	Novogene	Bioproject PRJNA438384
77	PVC2-P2	38519474	Hong Kong Water (22.34, 114.27)	MAR, 2017	Poly (vinyl chloride) panel	30 days	Novogene	Bioproject PRJNA438384
78	SS1	47084258	Hong Kong Water (22.34, 114.27)	MAR, 2017	Stainless steel panel	30 days	Novogene	Bioproject PRJNA438384
79	SS2	41032558	Hong Kong Water (22.34, 114.27)	MAR, 2017	Stainless steel panel	30 days	Novogene	Bioproject PRJNA438384
80	TI1	50880289	Hong Kong Water (22.34, 114.27)	MAR, 2017	Titanium panel	30 days	Novogene	Bioproject PRJNA438384

81	TI2	46151354	Hong Kong Water (22.34, 114.27)	MAR, 2017	Titanium panel	30 days	Novogene	Bioproject PRJNA438384
82	Beach1	85649091	Hong Kong Water (22.34, 114.27)	MAY, 2017	Rock panel	>30 days	Novogene	Bioproject PRJNA438384
83	Beach2	91839295	Hong Kong Water (22.34, 114.27)	MAY, 2017	Rock panel	>30 days	Novogene	Bioproject PRJNA438384
84	Rock1	81646889	Hong Kong Water (22.34, 114.27)	MAY, 2017	Rock panel	>30 days	Novogene	Bioproject PRJNA438384
85	Rock2	91212169	Hong Kong Water (22.34, 114.27)	MAY, 2017	Rock panel	>30 days	Novogene	Bioproject PRJNA438384
86	B-YSO	34941897	Yung Shu O Bay (22.43, 114.28)	JUN, 2017	Petri dish	12 days	Novogene	Bioproject PRJNA438384
87	B-ZH1	84990111	Sourth China Sea (21.70, 114.35)	JUL,2017	Petri dish	12 days	Novogene	Bioproject PRJNA438384
88	B-ZH2	83106135	Sourth China Sea (21.70, 114.35)	JUL,2017	Petri dish	12 days	Novogene	Bioproject PRJNA438384
89	B-ZH3	86665311	Sourth China Sea (21.70, 114.67)	JUL,2017	Petri dish	12 days	Novogene	Bioproject PRJNA438384
90	B-ZH4	75094624	Sourth China Sea (21.70, 114.67)	JUL,2017	Petri dish	12 days	Novogene	Bioproject PRJNA438384
91	B-ZH5	74014639	Sourth China Sea (21.70, 114.67)	JUL,2017	Petri dish	12 days	Novogene	Bioproject PRJNA438384
92	B-ECS2	88247462	East China Sea (30.70, 122.82)	AUG, 2017	Petri dish	30 days	Novogene	Bioproject PRJNA438384

93	B-ECS3	81067129	East China Sea (30.70, 122.82)	AUG, 2017	Petri dish	30 days	Novogene	Bioproject PRJNA438384
94	B-ECS4	76308372	East China Sea (30.70, 122.82)	AUG, 2017	Petri dish	30 days	Novogene	Bioproject PRJNA438384
95	B-ECS5	112505394	East China Sea (30.70, 122.82)	AUG, 2017	Petri dish	30 days	Novogene	Bioproject PRJNA438384
96	B-ECS6	102795008	East China Sea (30.70, 122.82)	AUG, 2017	Petri dish	30 days	Novogene	Bioproject PRJNA438384
97	B-ECS7	90909725	East China Sea (30.70, 122.82)	AUG, 2017	Petri dish	30 days	Novogene	Bioproject PRJNA438384
98	B-SY2	67949094	Sourth China Sea (18.23, 109.49)	NOV, 2017	Rock panel	>30 days	Novogene	Bioproject PRJNA438384
99	B-SY4	62990991	Sourth China Sea (18.23, 109.49)	NOV, 2017	Rock panel	>30 days	Novogene	Bioproject PRJNA438384
100	B-SY5	69201283	Sourth China Sea (18.23, 109.49)	NOV, 2017	Rock panel	>30 days	Novogene	Bioproject PRJNA438384
101	B-SCS	68232726	Sourth China Sea (14.00, 116.00)	JAN, 2018	Petri dish	3 days	Novogene	Bioproject PRJNA438384

Seawater samples (current study).

**Sample No.**	**Seawater metagenomes**	**Total sequences (×2)**	**Sampling locations**	**Sampling time**	**Filter**	**Accession (in NCBI)**
1	W-RSA1	68254289	Red Sea (22.20, 39.03)	APR, 2016	0.1-μm	Bioproject PRJNA438384
2	W-RSA2	55444950	Red Sea (22.20, 39.03)	APR, 2016	0.1-μm	Bioproject PRJNA438384
3	W-RSA3	59163194	Red Sea (22.20, 39.03)	APR, 2016	0.1-μm	Bioproject PRJNA438384

4	W-RSB1	58836750	Red Sea (22.20, 39.04)	APR, 2016	0.1-μm	Bioproject PRJNA438384
5	W-RSB2	53528721	Red Sea (22.20, 39.04)	APR, 2016	0.1-μm	Bioproject PRJNA438384
6	W-RSB3	57498088	Red Sea (22.20, 39.04)	APR, 2016	0.1-μm	Bioproject PRJNA438384
7	W-RSC1	69334016	Red Sea (22.20, 39.05)	APR, 2016	0.1-μm	Bioproject PRJNA438384
8	W-RSC2	64409249	Red Sea (22.20, 39.05)	APR, 2016	0.1-μm	Bioproject PRJNA438384
9	W-RSC3	65344164	Red Sea (22.20, 39.05)	APR, 2016	0.1-μm	Bioproject PRJNA438384
10	W-RSiii1	41007351	Red Sea (22.20, 39.04)	JUN, 2016	0.1-μm	Bioproject PRJNA438384
11	W-RSiii2	47482205	Red Sea (22.20, 39.04)	JUN, 2016	0.1-μm	Bioproject PRJNA438384
12	W-RSiii3	37690420	Red Sea (22.20, 39.04)	JUN, 2016	0.1-μm	Bioproject PRJNA438384
13	W-HK1707	39326525	Hong Kong Water (22.34, 114.27)	JUL, 2017	0.1-μm	Bioproject PRJNA438384
14	W-HK17081	48059457	Hong Kong Water (22.34, 114.27)	AUG, 2017	0.1-μm	Bioproject PRJNA438384
15	W-HK17082	48904872	Hong Kong Water (22.34, 114.27)	AUG, 2017	0.1-μm	Bioproject PRJNA438384
16	W-HK1709	85745326	Hong Kong Water (22.34, 114.27)	SEP, 2017	0.1-μm	Bioproject PRJNA438384
17	W-HK17111	71698362	Hong Kong Water (22.34, 114.27)	NOV, 2017	0.1-μm	Bioproject PRJNA438384
18	W-HK17112	84074099	Hong Kong Water (22.34, 114.27)	NOV, 2017	0.1-μm	Bioproject PRJNA438384
19	W-HK17113	90820365	Hong Kong Water (22.34, 114.27)	NOV, 2017	0.1-μm	Bioproject PRJNA438384

20	W-HK17121	88080567	Hong Kong Water (22.34, 114.27)	DEC, 2017	0.1-μm	Bioproject PRJNA438384
21	W-HK17122	92780086	Hong Kong Water (22.34, 114.27)	DEC, 2017	0.1-μm	Bioproject PRJNA438384
22	W-RSO2	78807175	Yung Shu O Bay (22.43, 114.28)	JUN, 2017	0.1-μm	Bioproject PRJNA438384
23	W-RSO3	71283306	Yung Shu O Bay (22.43, 114.28)	JUN, 2017	0.1-μm	Bioproject PRJNA438384
24	W-ZH	86106610	Zhuhai Xiangzhou Bay (22.29, 113.59)	JUL, 2017	0.1-μm	Bioproject PRJNA438384
**Selected Tara metagenomes (Sunagawa et al., 2015)**						
**Sample No.**	**Metagenomes**	**Total sequences (×2)**	**Sampling locations**	**Accession (in INSDC)**		
1	IO-ARAB-1	125582958	Indian Ocean (23.82, 63.50)	ERR599143		
2	IO-EAFR-1	314731164	Indian Ocean (-29.50, 38.00)	ERR598970		
3	IO-EAFR-2	145100047	Indian Ocean (-35.17, 26.29)	ERR598979		
4	IO-EAFR-3	145714747	Indian Ocean (-22.34, 40.34)	ERR599012		
5	IO-EAFR-4	162387844	Indian Ocean (-15.34, 43.30)	ERR599057		

6	IO-EAFR-5	168192870	Indian Ocean (-17.02, 42.74)	ERR599058
7	IO-ISSG-1	90667820	Indian Ocean (-9.39, 66.42)	ERR594314
8	IO-ISSG-2	249646209	Indian Ocean (-9.39, 66.42)	ERR599019
9	IO-ISSG-3	202283992	Indian Ocean (-16.96, 53.98)	ERR599098
10	IO-MONS-1	195519241	Indian Ocean (0.00, 71.64)	ERR599054
11	IO-MONS-2	220252033	Indian Ocean (14.61, 69.98)	ERR599074
12	IO-MONS-3	200861532	Indian Ocean (6.00, 73.90)	ERR599141
13	IO-MONS-4	130920132	Indian Ocean (19.04, 64.49)	ERR599158
14	MS-MEDI-1	179380493	Mediterranean Sea (35.76, 14.26)	ERR598993
15	MS-MEDI-2	110583306	Mediterranean Sea (37.05, 1.94))	ERR315857
16	MS-MEDI-3	74783005	Mediterranean Sea (42.20, 17.72)	ERR315861
17	MS-MEDI-4	157929848	Mediterranean Sea (33.92, 32.90)	ERR315862

18	MS-MEDI-5	147450847	Mediterranean Sea (39.16, 5.92)	ERR594288
19	MS-MEDI-6	198608764	Mediterranean Sea (39.39, 19.39)	ERR599043
20	NAO-CARB-1	157141812	North Atlantic Ocean (25.53, -88.39)	ERR599136
21	NAO-GFST	176015464	North Atlantic Ocean (39.23, -70.04)	ERR598983
22	NAO-GUIA	171199015	North Atlantic Ocean (9.85, -80.05)	ERR599029
23	NAO-NAST-E-1	173346004	North Atlantic Ocean (36.55, -6.57)	ERR598955
24	NAO-NAST-E-2	198143365	North Atlantic Ocean (36.17, -29.02)	ERR598976
25	NAO-NAST-E-3	164620027	North Atlantic Ocean (43.68, -16.83)	ERR599078
26	NAO-NAST-W-1	178945938	North Atlantic Ocean (34.11, -49.92)	ERR598963
27	NAO-NAST-W-2	169471567	North Atlantic Ocean (34.67, -71.31)	ERR598968
28	NAO-NAST-W-3	173119198	North Atlantic Ocean (31.69, -64.25)	ERR599123
29	NAO-NAST-W-4	193884779	North Atlantic Ocean (35.93, -37.30)	ERR599170

30	NPO-PNEC-01	185571189	North Pacific Ocean (14.20, -116.63)	ERR598989
31	NPO-PNEC-02	171139183	North Pacific Ocean (6.33, -102.93)	ERR599030
32	RS-REDS-01	34486935	Red Sea (27.16, 34.84)	ERR598969
33	RS-REDS-02	48683248	Red Sea (23.36, 37.22)	ERR599041
34	RS-REDS-03	85133875	Red Sea (21.95, 38.25)	ERR599049
35	RS-REDS-04	39588227	Red Sea (18.40, 39.88)	ERR598959
36	SAO-BENG-1	174220439	South Atlantic Ocean (-32.24, 17.71)	ERR594313
37	SAO-BENG-2	174320759	South Atlantic Ocean (-32.24, 17.71)	ERR594325
38	SAO-BENG-4	39041527	South Atlantic Ocean (-32.24, 17.71)	ERR598994
39	SAO-BENG-5	36385625	South Atlantic Ocean (-34.94,17.92)	ERR598973
40	SAO-FKLD	82951389	South Atlantic Ocean (-47.19, -58.29)	ERR599009
41	SAO-SATL-01	158033031	South Atlantic Ocean (-20.94, -35.18)	ERR594286
42	SAO-SATL-02	178203533	South Atlantic Ocean (-31.03, 4.67)	ERR594297
43	SAO-SATL-03	194974025	South Atlantic Ocean (-20.94, -35.18)	ERR594310

44	SAO-SATL-04	169647248	South Atlantic Ocean (-31.03, 4.67)	ERR594318
45	SAO-SATL-05	143741285	South Atlantic Ocean (-30.14, -43.29)	ERR594332
46	SAO-SATL-06	178378254	South Atlantic Ocean (-20.41, -3.18)	ERR594335
47	SAO-SATL-07	161070777	South Atlantic Ocean (-30.14, -43.29)	ERR594340
48	SAO-SATL-08	192910842	South Atlantic Ocean (-20.41, -3.18)	ERR594349
49	SAO-SATL-09	175664334	South Atlantic Ocean (-8.78, -17.91)	ERR598984
50	SAO-SATL-10	206080531	South Atlantic Ocean (-30.14, -43.29)	ERR599006
51	SAO-SATL-11	200803577	South Atlantic Ocean (-20.94, -35.18)	ERR599126
52	SAO-SATL-12	88675089	South Atlantic Ocean (-20.41, -3.18)	ERR599165
53	SAO-SATL-13	74463747	South Atlantic Ocean (-31.03, 4.67)	ERR599174
54	SO-ANTA-1	33639827	Southern Ocean (-60.23, -60.65)	ERR598945
55	SO-ANTA-2	37811950	Southern Ocean (-62.04, -49.53)	ERR599176

56	SPO-CHIL-1	137491742	South Pacific Ocean (-34.06, -73.11)	ERR599064
57	SPO-SPSG-01	79690789	South Pacific Ocean (-9.15, -140.52)	ERR594287
58	SPO-SPSG-02	82811132	South Pacific Ocean (-9.00, -139.20)	ERR594292
59	SPO-SPSG-05	169274791	South Pacific Ocean (-21.15, -104.79)	ERR599024
60	SPO-SPSG-03	230009431	South Pacific Ocean (-32.80, -87.07)	ERR599050
61	SPO-SPSG-05	186567128	South Pacific Ocean (-13.00, -95.98)	ERR599169
62	SPO-SPSG-04	126571370	South Pacific Ocean (-25.81, -111.72)	ERR599120
63	SPO-SPSG-08	188910040	South Pacific Ocean (-29.72, -101.16)	ERR598967
64	SPO-SPSG-09	160898544	South Pacific Ocean (-2.01, -84.59)	ERR599039
65	SPO-SPSG-10	190182767	South Pacific Ocean (16.96, -100.63)	ERR599077
66	SPO-SPSG-06	133022760	South Pacific Ocean (-9.00, -139.20)	ERR598307
67	SPO-SPSG-07	199504187	South Pacific Ocean (-23.28, -129.39)	ERR598954
